# Prediction of bacteremia in emergency department patients with suspected infection: an external validation of a clinical decision rule

**DOI:** 10.1186/cc12923

**Published:** 2013-11-05

**Authors:** Marie K Jessen, Julie Mackenhauer, Anne Mette Sondrup Wulff Hvass, Simon Skibsted, Hans Kirkegaard, Henrik C Schønheyder, Nathan I Shapiro

**Affiliations:** 1 Research Center for Emergency Medicine, Aarhus University Hospital, Aarhus, Denmark; 2 Department of Infectious Disease, Aarhus University Hospital, Aarhus, Denmark; 3 Research Department of Emergency Medicine, Beth Israel Deaconess Medical Center, Boston, MA, USA; 4 Department of Clinical Microbiology, Aalborg University Hospital, Aarhus University Hospital, Aalborg, Denmark

## Background

Bacteremia is a common clinical condition with an incidence of approximately 140 to 160 per 100,000 person-years. Since sepsis is a time-critical diagnosis, identification of emergency department (ED) patients at risk of bacteremia is therefore a priority. The study objective was to validate a previously published clinical decision rule for predicting a positive blood culture in ED patients with suspected infection based on minor criteria, major criteria and a total score [1].

## Materials and methods

This was a retrospective matched cohort study, set in a large urban academic tertiary ED at Aarhus University Hospital, Aarhus, Denmark with approximately 56,000 patient visits annually. Adult ED patients with blood cultures obtained from 1 January through 31 December 2011. ED patients with blood culture-confirmed bacteremia were matched 1:3 to patients with negative cultures. The outcome was true bacteremia. Features of the clinical history, co-morbid illnesses, physical observations and laboratory tests were used to evaluate the performance of the clinical decision rule including calculation of the total score (Table 1). We report operating characteristics and the summary *c*-statistic for the decision rule.

**Table 1:** Decision rule

Major criteria	Minor criteria (1 point each)
Suspected endocarditis (3 points)	Age >65 years
Temperature >39.4°C (103.0°F) (3 points)	Temperature 38.3 to 39.3°C
Indwelling vascular catheter (2 points)	Chills
	Vomiting
	Hypotension (systolic blood pressure <90 mmHg)
	White blood cell count >18,000 cells/mm^3^
	Bands >5% (in our setting, immature cells >0.5%)
	Platelets <150,000 cells/mm^3^
	Creatinine >2.0 mg/dl (177 µl/l)

A blood culture is indicated by the rule if at least one major criterion or two minor criteria are present. Otherwise, cultures may be omitted. Points used to calculate the total score.

## Results

**Figure 1:**
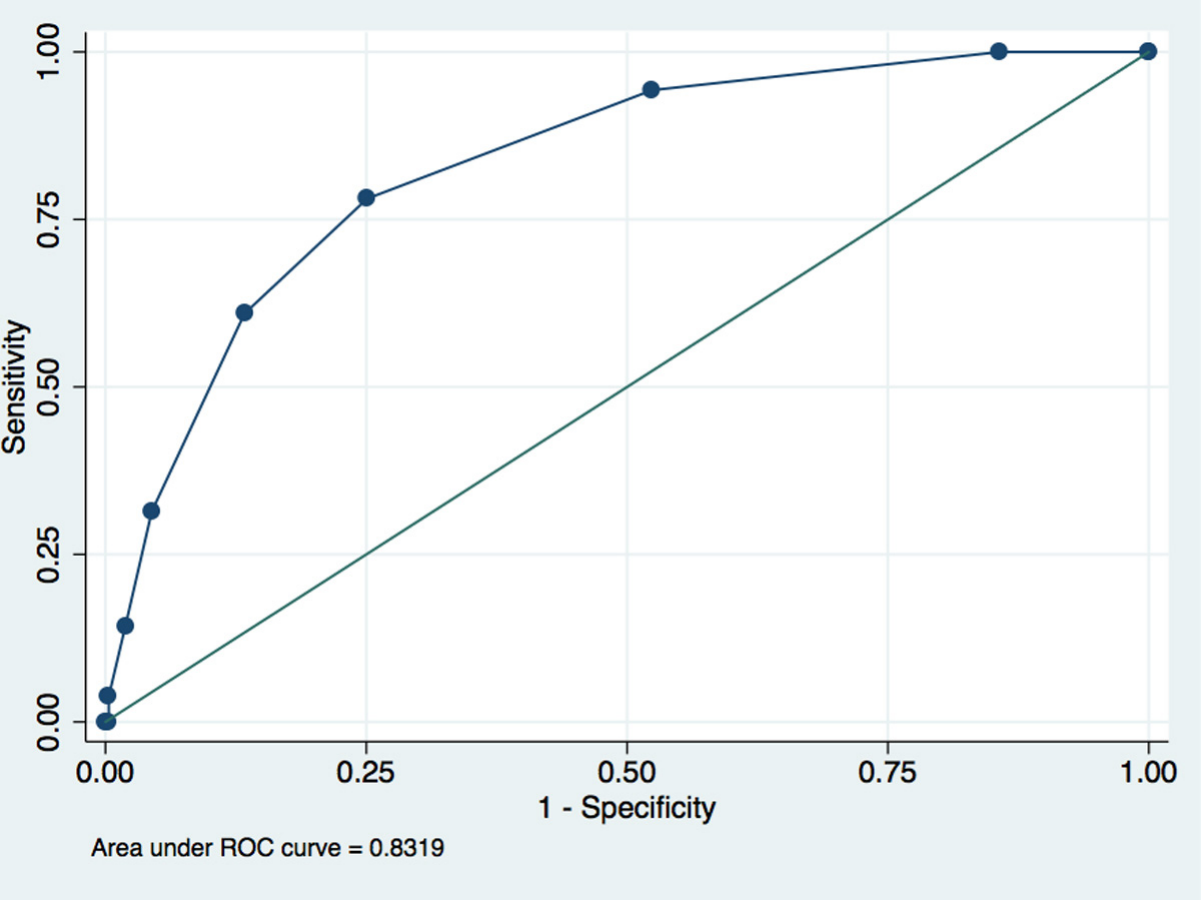
**Receiver operating characteristics curve (ROC) for external validation of the bacteremia prediction rule, calculated using the total score**.

Among 1,526 patients, 105 (6.9%) patients were classified with true bacteremia. The sensitivity of the prediction rule was 94% (95% confidence interval (CI) 88 to 98%) and specificity 48% (95% CI 42 to 53%). Positive and negative predictive values were 37% (95% CI 32 to 44%) and 96% (95% CI 92 to 99%), respectively. The area under the receiver-operating characteristics curve was 0.83 ± 0.02 standard error (Figure 1).

## Conclusions

The clinical decision rule performed well in our ED setting and is likely to be a useful supplement to clinical judgment.
